# Neonatal Intensive Care and Child Psychiatry Inpatient Care: Do Different Working Conditions Influence Stress Levels?

**DOI:** 10.1155/2013/761213

**Published:** 2013-06-27

**Authors:** Evalotte Mörelius, Per A. Gustafsson, Kerstin Ekberg, Nina Nelson

**Affiliations:** ^1^Division of Health, Activity, and Care, Department of Social and Welfare Studies, Faculty of Health Sciences, Linköping University, 601 74 Norrköping, Sweden; ^2^Division of Child and Adolescent Psychiatry, Department of Clinical and Experimental Medicine, Faculty of Health Sciences, Linköping University, 581 85 Linköping, Sweden; ^3^Department of Medical and Health Sciences, National Centre for Work and Rehabilitation, Faculty of Health Sciences, Linköping University, 581 85 Linköping, Sweden; ^4^Division of Pediatrics, Department of Clinical and Experimental Medicine, Faculty of Health Sciences, Linköping University, 581 85 Linköping, Sweden

## Abstract

*Introduction*. Nurses often experience work-related stress. High stress can negatively affect job satisfaction and lead to emotional exhaustion with risk of burnout. *Aim*. To analyse possible differences in biological stress markers, psychosocial working conditions, health, and well-being between nurses working in two different departments. *Methods*. Stress was evaluated in nurses working in a neonatal intensive care unit (NICU) (*n* = 33) and nurses working in a child and adolescent psychiatry inpatient ward (CAP) (*n* = 14) using salivary cortisol and HbA1c. Salivary cortisol was measured three times a day on two consecutive days during two one-week periods, seven weeks apart (= 12 samples/person). Psychosocial working conditions, health, and well-being were measured once. *Results*. NICU nurses had better social support and more self-determination. CAP nurses had a lower salivary cortisol quotient, poorer general health, and higher client-related burnout scores. *Conclusion*. When comparing these nurses with existing norm data for Sweden, as a group their scores reflect less work-related stress than Swedes overall. However, the comparison between NICU and CAP nurses indicates a less healthy work situation for CAP nurses. *Relevance to Clinical Practice*. Healthcare managers need to acknowledge the less healthy work situation CAP nurses experience in order to provide optimal support and promote good health.

## 1. Introduction

Compared with outpatient nursing, perceived stress is higher among nurses working in internal medicine, intensive care, accident and emergency wards, and in paediatrics [1]. Neonatal intensive care unit (NICU) nurses are an example of a nursing discipline commonly exposed to high work-related stress [2–4]. Nurses working with psychiatrically ill children and adolescents are exposed to emotional strain, although of a somewhat different nature than the strain faced by nurses working with life-threatening conditions, as in the NICU. Some studies confirm this finding [5–7], but we could not find any studies specifically addressing differences in stress between NICU nurses and nurses involved in inpatient child and adolescent psychiatric care. 

The stress system coordinates the generalised stress response, which can be defined as the physiological response to environmental demands placed upon the individual [8]. Activation of the stress system initiates behavioural and peripheral changes in order to improve homeostasis. Cortisol modulates the stress response by acting on the hypothalamus to inhibit continued release of corticotropin-releasing hormone (CRH) [9]. Salivary cortisol is an example of an objective representative of the stress reaction [10]. Under normal conditions, cortisol levels vary throughout the day, with high concentrations in the morning followed by a progressive decline towards low levels in the evening. Chronic stress, however, may flatten the normal cortisol circadian pattern, resulting in higher cortisol levels in the evening [11]. Cortisol elevation for the purpose of releasing energy is beneficial from a short-term perspective, but it may become harmful if the elevation becomes chronic [10]. Chronic stress alters responsiveness to acute stressors [12], resulting in loss of appropriate responses (allostasis) to environmental demands [10, 13, 14]. 

Work-related stress, discomfort, and helplessness are related to elevated biological responses resulting from overactivation of the sympathoadrenal and hypothalamus-pituitary-adrenal (HPA) axis [15, 16]. Emotional stress and depression may negatively affect work satisfaction among nurses [17]. Gillespie and Melby (2003) found that nurses working on an internal medicine ward experienced higher levels of emotional exhaustion than nurses working in the accident and emergency department [18]. In a study involving 93 nurses working in acute mental health in the UK, approximately half showed signs of emotional exhaustion [19]. Work-related stress with regard to job loading, role conflict, organizational interaction, and interpersonal relationships correlates with occupational burnout and may be a significant factor influencing new nurses to quit their jobs [20, 21]. In addition, workload among experienced healthcare workers has been shown to significantly correlate with higher cortisol levels, particularly in the evening [22–24]. Neonatal intensive care may be considered stressful; stress-related cortisol elevation is common among nurses working in neonatal or paediatric intensive care units according to Fujimaru et al. [25]. Therefore, measuring cortisol levels may illuminate working conditions in this type of environment [5]. 

Social support may have a buffering effect; higher levels of social support from coworkers are associated with lower levels of emotional exhaustion [19]. A qualitative study among intensive care staff in Sweden found that healthcare workers were empowered both by internal processes, such as feelings of doing good, increased self-esteem/self-confidence, and increased knowledge and skills, and by external processes, such as nurturing encounters, good teamwork, and supportive atmosphere [26]. Hallberg (1994) found that work satisfaction among nurses in a paediatric psychiatric care ward improved (1) when they were understood and understood others, which led to improved cooperation and self-confidence, and (2) as a result of a broader and better knowledge base, which increased and improved goal-oriented and active nursing interventions in clinical practice. Nursing satisfaction increased significantly in regard to responsibility, organisation, quality of care, cooperation, and comfort level in the work group [27]. 

This first aim was to analyse possible differences in biological stress markers, psychosocial working conditions, and health and well-being between nurses working in a neonatal intensive care unit and nurses working in a child and adolescent psychiatry inpatient ward. The second aim was to analyse possible correlations between biological stress markers and psychosocial working conditions and health and well-being, respectively.

## 2. Materials and Methods

### 2.1. Design

An exploratory, prospective design was used to compare nurses' health, stress reactions, and work conditions in two different departments.

### 2.2. Participants

The study was conducted in two departments at a university hospital in one of six health care regions in Sweden: (1) a level III neonatal intensive care unit (NICU) and (2) a child and adolescent psychiatry inpatient ward (CAP). These two wards are the only ones providing neonatal intensive care and child and adolescent psychiatry inpatient care, respectively, in this healthcare region. Thirty-three out of 56 NICU nurses and 14 out of 18 CAP nurses participated in the study.

#### 2.2.1. Neonatal Intensive Care Unit (NICU)

The NICU has nine neonatal intensive care beds and another eight bed for neonatal care. Most parents stay with their infants round the clock, in the patient room or in special parent rooms located on the ward. Workload is the same 24 hours a day. NICU work is characterised by acute care: rapid assessments followed by immediate interventions. Intervention results can often be immediately evaluated, providing direct feedback. The scenarios are “here and now”. The nurses have well-defined duties and work with the parents for the benefit of the infants. Although NICU situations sometimes end in sorrow, happiness and satisfaction are more typical of outcomes (personal communication: Marie Hassel). 

#### 2.2.2. Child and Adolescent Psychiatry Inpatient Ward (CAP)

At the time of this study, CAP had four beds for child psychiatric care. Parents may stay overnight in the same room with their children, but because most patients are adolescents, this is uncommon. Workload is usually higher during the day than at night, when patients usually are asleep. Typically, inpatient CAP care is indicated for suicidal adolescents to prevent patients from harming themselves. The social circumstances are often difficult: children may come from broken homes and foster care, or they may have been victims of abuse, and so forth. The most problematic aspects of the child's situation often lie beyond the realm of solutions available to staff—encouraging a mother to leave her abusive husband, finding a new foster home, and speeding up court proceedings in sexual abuse cases. An important aspect of work is to foster patient trust in the nurse. To achieve such a trusting relationship, nurses must apply a myriad of interpersonal skills. Thus, the working situation is less governed by general instructions and less welldefined than in the NICU (personal communication: Malin Mobom). 

#### 2.2.3. Study Sequence

The research coordinator provided oral and written information about the study to all nurses working in the included wards (nurses, registered nurses, and nurse specialists). Pregnant nurses and nurses regularly taking medications known to affect the hypothalamus-pituitary axis, such as levothyroxine, were excluded (*n* = 3). All participants provided informed consents. Saliva samples were collected during two one-week periods, seven weeks apart. During each collection week, saliva samples were taken at 7 a.m., 4 p.m., and 10 p.m. on two consecutive days (12 saliva samples per individual). Saliva samples were obtained at the same time, regardless of individual work schedule, which means that samples were taken both at home and at work, depending on the location of the participant. A mood scale questionnaire was completed in conjunction with each 4 p.m. saliva sample. A blood sample for HbA1c was obtained along with demographic information, and the instruments described below (JD-C, JD-C-S, PEI, CBI, and SF-36) were completed once during the first study week.

### 2.3. Measures

#### 2.3.1. Biological Markers****

*Salivary Cortisol.* Saliva was collected using the Salivette test tubes (Sarstedt, Rommelsdorf, Nümbrecht, Germany). Since several components can confound salivary cortisol measurements, participants were told not to eat, drink, smoke or brush their teeth one hour before sampling. After collection, the saliva was stored at the university hospital at −20°C pending analysis. All samples were analysed with a modified commercial radioimmunoassay (RIA) from Diagnostic Products Corporation, CA, USA [29]. Inter- and intra-assay coefficients of variation were 8.3% (<10 nmol/L) and 5.1% (>10 nmol/L) and 4.3% (<10 nmol/L) and 3.6% (>10 nmol/L), respectively. 

*HbA1c.* HbA1c can be viewed as a stress marker [30]. As described by Kawakami et al. [31], greater work stress and lower social support in the workplace may be associated with increased HbA1c levels. Blood samples for HbA1c analysis were sent to the Department of Clinical and Laboratory Investigation at the University Hospital. HbA1c was analysed using a photometric method and compared with a Swedish standard, external quality assurance in laboratory medicine in Sweden, EQUALIS AB.

#### 2.3.2. Psychosocial Working Conditions****

*Job Demand-Control Model (JD-C).* The Job Demand-Control model [32] focuses on two dimensions of the work environment: job demands and job control. Job demands pertain to workload and have been operationalised mainly in terms of time pressure and role conflict [33]. Job control, also called decision latitude, refers to a person's ability to control his or her work activities. Decision latitude includes two components: skill discretion and decision authority. The model consists of eleven items rated on a 4-point Likert scale, ranging from “strongly agree” (1) to “strongly disagree” (4). Job strain is calculated as the quotient between demands and control. 

*Social Support (JD-C-S).* The social support scale in the JD-C is used to measure social support in the workplace. A summary index is calculated as the average score of the six items and ranges from 6 to 24 [34]. 

*Psychological Empowerment Instrument (PEI)*. Spreitzer's 12-item psychological empowerment instrument (PEI) was used to assess empowerment [35]. The instrument has been translated and tested for psychometric properties in a Swedish sample [36]. The instrument has four subscales measuring perceived meaning, competence, self-determination, and impact at work. The score of the four subscales ranges from 1 (lowest level of empowerment) to 7 (highest level of empowerment).

#### 2.3.3. Health and Well-Being****

*The Copenhagen Burnout Inventory (CBI).* The Copenhagen Burnout Inventory (CBI) was used to measure burnout [37]. The CBI focuses on exhaustion and is divided into three scales: personal burnout, work-related burnout, and client-related burnout. These different scales consist of six, seven, and six items, respectively. Each item has a 5-point Likert response scale ranging from “always” to “never/almost never” and from “a very high degree” to “a very low degree”. Summary scores are calculated and converted into a score ranging from 0 (lowest possible degree of burnout) to 100 (highest degree of burnout). The originators apply a cut-off for clinical burnout at an average subscale score of 50 and above [37, 38]. 

*Quality of Life, SF-36.* The SF-36, or Medical Outcomes Study (MOS) 36-Item Short-Form Health Survey, is a multipurpose health survey. The SF-36 is designed to be a generic indicator of health status for use in population surveys and to be applicable in a wide range of types and severities of health conditions and in a variety of clinical and nonclinical populations [39]. The SF-36 generates a health profile based on eight scale scores: physical functioning (PF), role limitations due to physical health problems (RP), bodily pain (BP), general health perceptions (GH), vitality, energy or fatigue (VT), social functioning (SF), role limitations due to emotional problems (RE), and mental health (MH). The first four scales can be regarded as measuring primarily physical aspects of health and the latter four mainly mental aspects, although GH and VT measure health in more general terms [40]. All scales range from 0 to 100, where high scores correspond to better health. Swedish population norm values were used for comparison when assessing SF-36 scores [28].

*Mood Scale Questionnaire.* The mood scale was used to measure the bipolar dimensions of mood [41]. The instrument consists of 71 adjectives measured on a 4-point scale (1= it definitely disagrees with what I feel right now and 4= it definitely agrees with what I feel right now). Examples of adjectives are “secure”, “relaxed”, “happy”, “energetic”, and “sociable”. The adjectives are divided into six dimensions (control, calmness, social orientation, pleasantness, activation, and extraversion). The dimensions are analysed separately and are also used to generate a total sum mean score. A total of four mood scale questionnaires per individual were completed [41].

### 2.4. Ethical Considerations

The local ethics committee at the university approved the study, and the informed consent was obtained from all participating nurses (D# M173-4).

### 2.5. Data Analysis

Data were analysed using SPSS statistical software (version 17.0). The salivary cortisol quotient was calculated by dividing the morning value with the evening value. Salivary cortisol levels and data from instruments (JD-C, JD-C-S, PEI, CBI, SF-36, and mood scale) are presented as mean and SD; potential differences between groups (NICU and CAP nurses) were tested with independent sample *t*-test. Pearson's correlation was used to calculate possible correlations. Most obtained data mentioned above were normally distributed (the Kolmogorov Smirnov test); if not, analyses were recalculated using nonparametric statistics. Since all recalculations yielded the same significances nonparametric analyses are not presented. The Chi-square test was used to test potential differences in demographic and descriptive characteristics. Findings were considered statistically significant when *P* < 0.05. No power calculation was made because all nurses working with neonatal intensive care and child and adolescent psychiatry inpatient care at the university hospital were invited to participate in the study.

## 3. Results

No significant differences were observed in demographics between the groups, with the exception that significantly more NICU nurses were nonsmokers compared with CAP nurses (Table 1).

### 3.1. Biological Markers

**Figure 1:**
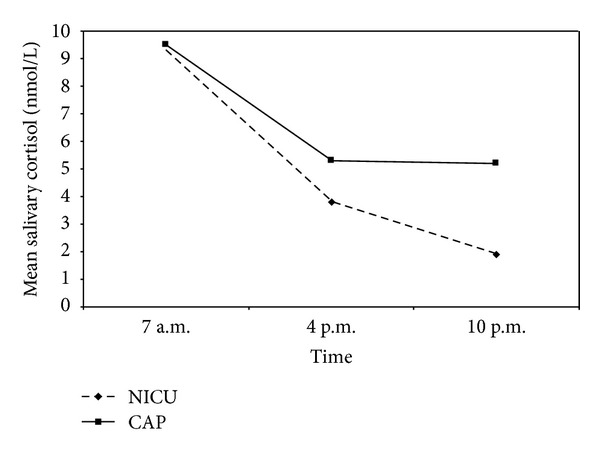
Mean salivary cortisol at three time points for nurses from the neonatal intensive care unit (NICU) and the child and adolescent psychiatry inpatient ward (CAP), respectively.

A total of 372 salivary cortisol samples from NICU nurses and 116 from CAP nurses were collected, analysed, and included in the statistical analyses. No significant difference in individual salivary cortisol levels was found between the first and second weeks of sampling, neither for 7 a.m., 4 p.m., nor 10 p.m. A mean value from the different weeks could therefore be calculated for each participant and sample-time for further analysis. Mean (SD) salivary cortisol level at 7 a.m. was 9.3 (5.6) nmol/L for NICU nurses and 9.5 (10.2) nmol/L for CAP nurses (n.s.). Mean (SD) salivary cortisol level at 4 p.m. was 3.8 (3.0) nmol/L for NICU nurses and 5.3 (6.7) nmol/L for CAP nurses (n.s.). Mean (SD) salivary cortisol level at 10 p.m. was 1.9 (2.4) nmol/L for NICU nurses and 5.2 (10.6) nmol/L for CAP nurses (*P* < 0.001) (Figure 1). NICU nurses had a significantly higher morning/evening quotient than CAP nurses. The mean (SD) salivary cortisol quotient was 10.1 (9.5) for NICU nurses and 4.7 (4.4) for CAP nurses (*P* < 0.001). Thirty-one (94%) HbA1c samples were collected and analysed from NICU nurses and eight (57%) from CAP nurses. No significant difference in HbA1c was found between the groups. Mean (SD) HbA1c was 4.4% (0.3) for NICU nurses and 4.2% (0.3) for CAP nurses.

Statistically significant positive correlations were found between salivary cortisol quotient and social support (JD-C-S) (*r* = 0.37; *P* = 0.01), as well as between salivary cortisol quotient and self-determination (PEI) (*r* = 0.30; *P* = 0.045). No correlation was found between salivary cortisol quotient and instruments measuring either health or well-being.

### 3.2. Psychosocial Working Conditions

No significant differences were found between the two groups of nurses regarding JD-C; demand index score, control index score, or job strain. However, NICU nurses had significantly higher JD-C-S index score compared with CAP nurses (*P* = 0.04) (Table 2). NICU nurses also had a significantly higher score on the PEI subscale self-determination compared with CAP nurses (*P* = 0.003) (Table 2).

### 3.3. Health and Well-Being

**Figure 2:**
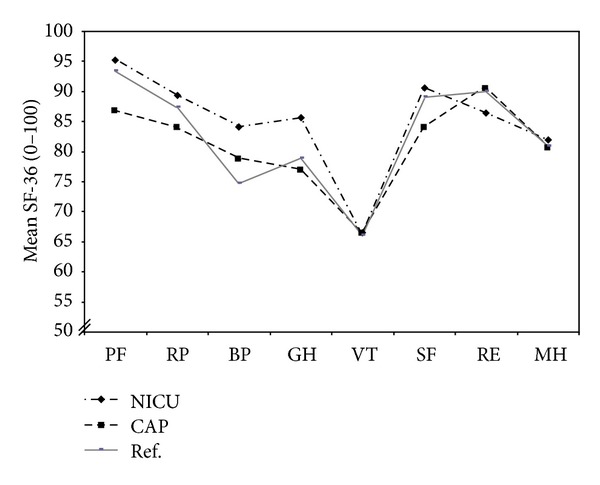
Mean values for eight subscales of SF-36 (see below) for nurses from the neonatal intensive care unit (NICU) and the child and adolescent psychiatry inpatient ward (CAP), respectively, compared with a reference sample of Swedish women (*n* = 515) working in health care [28].

NICU nurses had significantly lower client-related burnout scores on the CBI scale compared with CAP nurses (*P* = 0.035). No significant differences in personal or work-related burnout scores were found between the two groups of nurses (Table 2), nor were any significant differences found in SF-36 total sum score. However, NICU nurses had significantly higher scores, indicating better health, in the subscales physical functioning (PF) and general health perception (GH). NICU nurses also scored slightly better in PF and GH, and CAP nurses scored slightly lower in PF, compared with a reference population of 515 female healthcare employees in southeast Sweden [28] (Figure 2). No significant differences were found in total mood scale score (Table 2) or in the separate dimensions of the mood scale between NICU nurses and CAP nurses on any occasion (data not shown).

## 4. Discussion

The differences in salivary cortisol indicate somewhat higher biological stress levels in CAP nurses, that is, higher evening cortisol and lower morning/evening quotient. Lower morning/evening quotients indicate a flattened circadian pattern, as previously described in relation to high or chronic stress [11]. The findings are similar to those of Wingenfeld et al. (2009), who found higher cortisol release throughout the day in subjects with higher burnout scores. High mental workload without the ability to actively influence and control the situation may cause discomfort, resignation, and increased stress hormones, especially cortisol [15]. A higher salivary cortisol quotient correlates significantly with higher “self-determination” and better “social support”. These findings are plausible since empowerment and social support have both previously been shown to buffer against stress [19, 42, 43].

Overall, the two groups of nurses did not report substantial work-related stress, and most measurements showed no differences between the staff at the two workplaces. But some differences were present, all seemingly pointing to less work-related stress among NICU nurses whose working conditions are characterised by acute and rapid care. CAP nurses scored lower on “social support”, possibly indicating more conflicts and reflecting the special working conditions arising from milieu therapy. When caring for young inpatients, nurses are expected to maintain rules and draw the line for unacceptable behaviour, while simultaneously maintaining a caring and supportive relationship. Walking such a fine line is open to subjective evaluation, and different viewpoints may easily lead to conflict among staff, as well as between staff and patients. Also, NICU nurses scored higher on “self-determination” (i.e., a feeling of being in charge and able to initiate and control actions). One interpretation is that the organisation and job description for each occupation in the NICU ward are distinct, defined, and hierarchical with clearly specified responsibilities and mandates for nurses, while in the CAP ward all staff work under a more equal and less hierarchical structure, with less clearly defined and more ambiguous duties. Consequently, actions intended to stabilise and solve problems are often more highly scrutinised in the CAP. NICU nurses usually also receive direct feedback on their actions, while CAP nurses may have to wait weeks or months before they are able to assess the possible impact of their care. Still, it is important to consider that the scores for both groups indicate less work-related stress than for comparable reference populations, even though significantly less stress was found among NICU nurses.

CAP nurses reported a higher client-related (but not work-related) burnout score on the CBI, which may indicate that involvement in the difficult psychiatric and psychosocial situation of their clients poses a larger burden [44]. Nurses and staff working in acute mental health settings have rated how they are often exposed to violent and aggressive behaviours from patients and that it may be difficult to leave their work behind when at home [45, 46]. NICU nurses are also involved with their clients, but with one important difference: parents of NICU infants are present as primary caregivers on the ward most of the time [47]. The CAP-ward children are often in perpetual conflict with their parents, who are neither present nor able to accept full parental responsibility.

CAP nurses reported lower “physical functioning” and “general health perception” compared with NICU nurses and lower “physical functioning” compared with other female healthcare workers [28]. However, no correlations were found between salivary cortisol quotient and either “physical functioning” or “general health perception”. Long-term stress is known to negatively affect general health [10]; that is why it is important to identify nurses experiencing high work-related stress to prevent future health problems.

Kawakami et al. [31] previously reported that greater work-related stress and lower social support in the workplace may be associated with increased concentrations of HbA1c. However, our study was unable to confirm these results, which may be explained by the limited number of participants and/or the fact that both groups scored relatively well in health and well-being. The relatively large and unbalanced dropout rate between groups can of course conceal a possible difference between groups, especially since the larger HbA1c dropout rate was in the CAP group with lower social support and self-determination along with differences in cortisol levels indicating higher biological stress. The lower number of HbA1c samples could possibly be explained by the necessity to leave a blood sample, a fact maybe more pronounced in a subgroup with higher overall stress load.

The sample is relatively small; thus, the statistical power to detect differences between personnel at the two wards was low. As shown, the number of NICU nurses was greater, but the proportion of participating CAP nurses was higher. Theoretically, both circumstances may affect the results due to possible selection bias. Also, smoking which showed a skewed representation (more among CAP nurses) may influence the salivary cortisol results [48]. However, omitting them from the analysis was not an option, since many smokers were found in this group. Notably, the science on if, how, and in what direction smoking affects cortisol levels is not consistent [49, 50], which is why we cannot draw any further conclusions on this matter. 

## 5. Conclusion

When comparing these nurses with existing norm data for Sweden, they score less work-related stress than Swedes overall, despite the obvious stressors they experience at work. However, when comparing NICU with CAP nurses, the work situation of CAP nurses appears to be less healthy, which indicates that different working conditions influence the stress levels. This should be kept in mind for the management of inpatient CAP care and when planning improvements to this type of hospital care. 

## Figures and Tables

**Table 1:** Demographic and descriptive characteristics of nurses from the neonatal intensive care unit (NICU) and the child and adolescent psychiatry inpatient ward (CAP), respectively.

	NICU *n* = 33 (%)	CAP *n* = 14 (%)	*P* value
Age group, years			n.s.
20–30	7 (21.2)	1 (7.1)	
31–40	6 (18.2)	3 (21.4)	
41–50	11 (33.3)	5 (35.7)	
51–60	9 (27.3)	5 (35.7)	
Years of training as a nurse			n.s.
1–5	7 (21.2)	3 (21.4)	
6–10	3 (9.1)	1 (7.1)	
11–15	3 (9.1)	2 (14.2)	
16–20	2 (6.1)	2 (14.2)	
>20	18 (54.5)	6 (42.8)	
Married or cohabiting	29 (88)	11 (78.5)	n.s.
Children living at home	16 (48)	8 (57)	n.s.
Do not smoke	28 (85)	7 (50)	0.005
Do not use snuff	32 (97)	13 (93)	n.s.

**Table 2:** Mean and standard deviations (SD) for the Job Demand-Control Model (JD-C and JD-C-S), the Psychological Empowerment Instrument (PEI), and the Copenhagen Burnout Inventory (CBI) for nurses from the neonatal intensive care unit (NICU) and the child and adolescent psychiatry inpatient ward (CAP), respectively.

	NICU *n* = 33 Mean (SD)	CAP *n* = 14 Mean (SD)	*P* value
JD-C, Demand	11.8 (1.2)	11.3 (1.0)	n.s.
JD-C, Control JD-C, Job strain	18.9 (1.9) 1.6 (0.5)	18.1 (1.8) 1.8 (0.4)	n.s. n.s.
JD-C-S, Social support	21.3 (2.4)	19.9 (2.0)	0.04
PEI, Meaning	6.0 (0.6)	5.9 (1.0)	n.s.
PEI, Competence	5.8 (0.6)	5.8 (0.8)	n.s.
PEI, Self-determination	4.8 (0.7)	4.2 (0.8)	0.003
PEI, Impact at work	4.3 (1.1)	4.6 (0.8)	n.s.
CBI, Personal-related burnout	32.4 (13.8)	32.4 (14.5)	n.s.
CBI, Work-related burnout	24.3 (9.9)	28.1 (16.3)	n.s.
CBI, Client-related burnout	13.3 (9.1)	22.9 (15.7)	0.035
